# Family reunion via error correction: an efficient analysis of duplex sequencing data

**DOI:** 10.1186/s12859-020-3419-8

**Published:** 2020-03-04

**Authors:** Nicholas Stoler, Barbara Arbeithuber, Gundula Povysil, Monika Heinzl, Renato Salazar, Kateryna D Makova, Irene Tiemann-Boege, Anton Nekrutenko

**Affiliations:** 1 Graduate Program in Bioinformatics and Genomics, The Huck Institutes for Life Sciences, The Pennsylvania State University, University Park, PA USA; 2 Department of Biology, The Pennsylvania State University, University Park, PA USA; 3 Institut für Biophysik, Johannes Kepler Universität, Linz, Österreich Austria; 4 Present Address: Institute for Genomic Medicine, Columbia University Irving Medical Center, New York, NY USA

**Keywords:** Duplex sequence, Low frequency variants, Barcodes, Error correction

## Abstract

**Background:**

Duplex sequencing is the most accurate approach for identification of sequence variants present at very low frequencies. Its power comes from pooling together multiple descendants of both strands of original DNA molecules, which allows distinguishing true nucleotide substitutions from PCR amplification and sequencing artifacts. This strategy comes at a cost—sequencing the same molecule multiple times increases dynamic range but significantly diminishes coverage, making whole genome duplex sequencing prohibitively expensive. Furthermore, every duplex experiment produces a substantial proportion of singleton reads that cannot be used in the analysis and are thrown away.

**Results:**

In this paper we demonstrate that a significant fraction of these reads contains PCR or sequencing errors within duplex tags. Correction of such errors allows “reuniting” these reads with their respective families increasing the output of the method and making it more cost effective.

**Conclusions:**

We combine an error correction strategy with a number of algorithmic improvements in a new version of the duplex analysis software, Du Novo 2.0. It is written in Python, C, AWK, and Bash. It is open source and readily available through Galaxy, Bioconda, and Github: https://github.com/galaxyproject/dunovo.

## Background

**Fig. 1:**
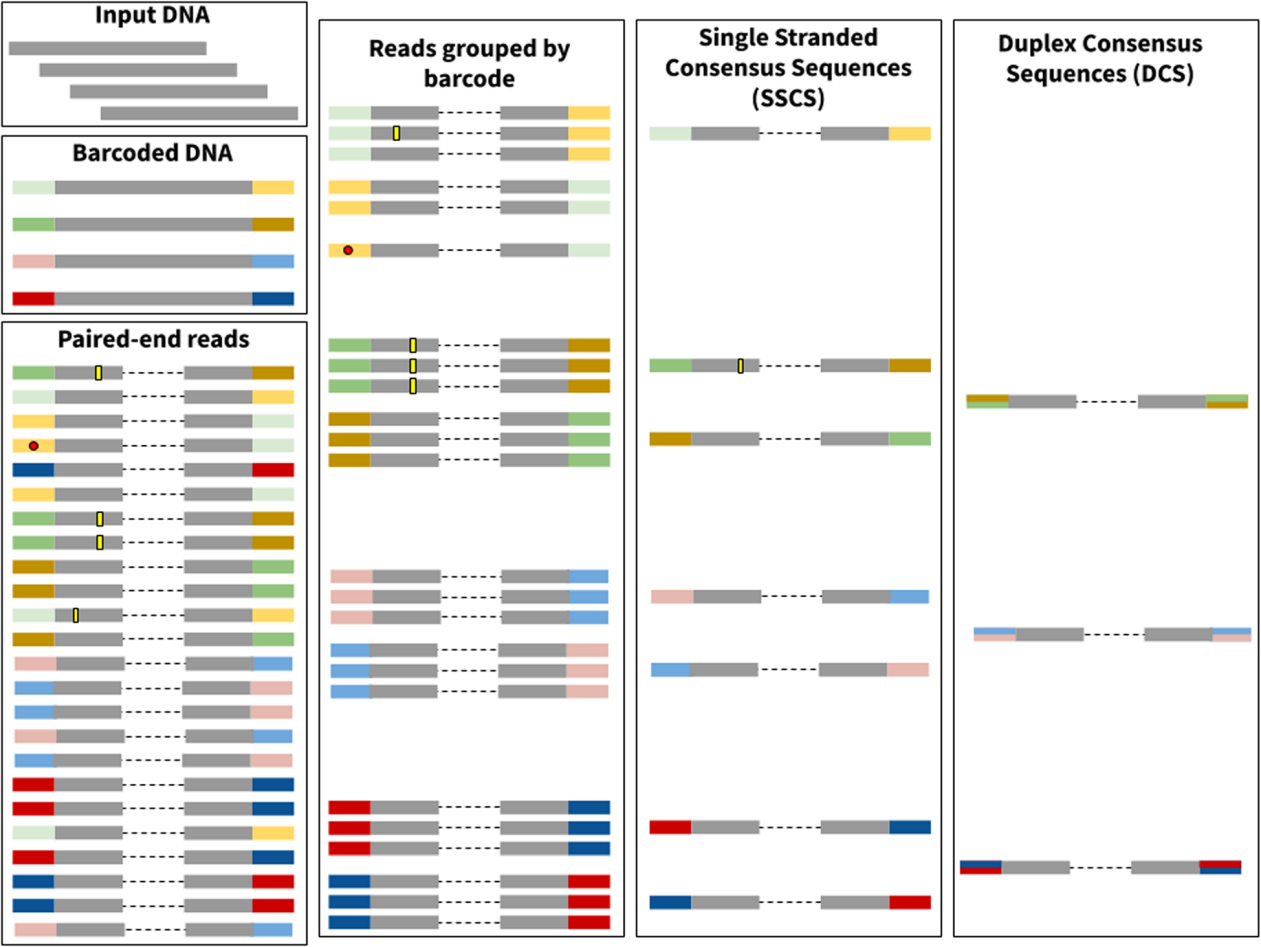
Effect of errors on the Duplex Sequencing procedure. Here input DNA is sheared and barcodes are ligated to the ends of the DNA molecules (colored rectangles in Barcoded DNA). After paired-end library preparation and sequencing each original molecules gives rise to multiple reads (Paired-end reads pane). This process also inadvertently generates sequencing errors represented by yellow rectangles and red circles. The yellow rectangles and red circles are used to depict errors arising inside read compartments corresponding to original DNA and adapters, respectively. Reads are then grouped by barcode to produce “*families*”. In this example each family is required to contain at least three reads. As shown here one of the reads contains an error (red circle) within the barcode. The error makes this particular barcode different from others. As a result it cannot be added into the family and remains a singleton (the error correction algorithm described here was developed specifically to correct such errors and allow singletons to be joined with their respective families). Each family is subsequently reduced into a Single Strand Consensus Sequence (SSCS) and each respective SSCS is merged with its counterpart from the opposite strand to generate Duplex Consensus Sequences (DCS)

Numerous, often clinically important, research scenarios require detection of sequence variants that are present in a minute fraction (10^− 5^–10^− 9^) of molecules under study. Examples include detection of cancer-related mutations in liquid biopsies, identification of fetal DNA in a mother’s bloodstream, assessing dynamics of the immune system, tracing mutational landscape of bacteria through the evolution of antibiotic resistance, studying genomic changes in viral pathogens and many others (for a comprehensive review see [7]). Conventional approaches, where a sample is sequenced and resulting reads are aligned against a reference genome to find differences, are ill suited for variants present at frequencies below 1% [6, 9]. A number of techniques has been developed to circumvent this issue with Duplex Sequencing (DS) being currently the most sensitive [7, 8]. DS is based on using unique tags (also called barcodes throughout this manuscript) to label individual molecules of the input DNA. During amplification steps that are required for the preparation of Illumina sequencing libraries, each of these molecules gives rise to multiple descendants. The descendants of each original DNA fragment are identified and grouped together using tags—one simply sorts tags in sequencing reads lexicographically and all reads containing the same tag are bundled into a *family*. These families (with at least three members or more) form single stranded consensus sequences (SSCS) for the forward or the reverse strand, respectively. Complementary SSCSs are then grouped to produce duplex consensus sequences (DCSs; see Fig. 1). A legitimate sequence variant is found in the majority of the reads within a family. In contrast, sequencing and amplification errors will manifest themselves as “polymorphisms” within a family and so can be identified and removed (yellow rectangles in Fig. 1).

Despite its power DS is a complex technique. Reliable identification of sequence variants requires each initial fragment to form a family with at least three members for each strand. To achieve this, it is necessary to precisely quantify the amount of input DNA during the library preparation step. Too much DNA results in small family sizes and makes variant identification impossible, while too little creates very large families at the expense of sequencing coverage. Furthermore, because DS barcodes are a part of the sequencing read, they accumulate PCR and sequencing errors. These errors prevent matching barcodes and therefore artificially split DS read families (red dots in Fig. 1) decreasing the efficiency of the procedure. In this manuscript we describe a new, efficient approach to the analysis of DS data that includes barcode error correction. It significantly improves the yield and performance of the technique. We also describe new quality control approaches designed to increase output of DS experiments.

## Results and discussion

### Datasets

To test our results we used two previously published datasets. The first dataset was produced by Schmitt et al. [9], who employed DS to identify a rare mutation at the *ABL1* locus responsible for resistance to a chronic myeloid leukemia therapeutic compound imatinib. The second dataset was produced by our group as a part of an experimental evolution study where DS was used to track frequencies of adaptive mutations in plasmid pBR322 [5].

### Barcode errors result in lost data

Typical DS tags are randomized 12-mers. Since each DNA fragment is labeled by two tags, one at each end, the theoretical upper bound for the number of unique combinations is 4^(12 + 12)^. However, the input DNA in a standard DS experiment contains ~ 10^6^ – 10^11^ molecules, creating a large tag-to-input excess (4^24^ ≫ 10^11^). Because of this excess it is highly unlikely to observe distinct input DNA molecules tagged by barcodes that are highly similar to each other. In fact, we can use this assumption to identify barcodes containing sequencing errors: barcodes that differ from each other by just a few nucleotides are likely descendants of the same original sequence with differences introduced by PCR and/or sequencing errors.

**Fig. 2:**
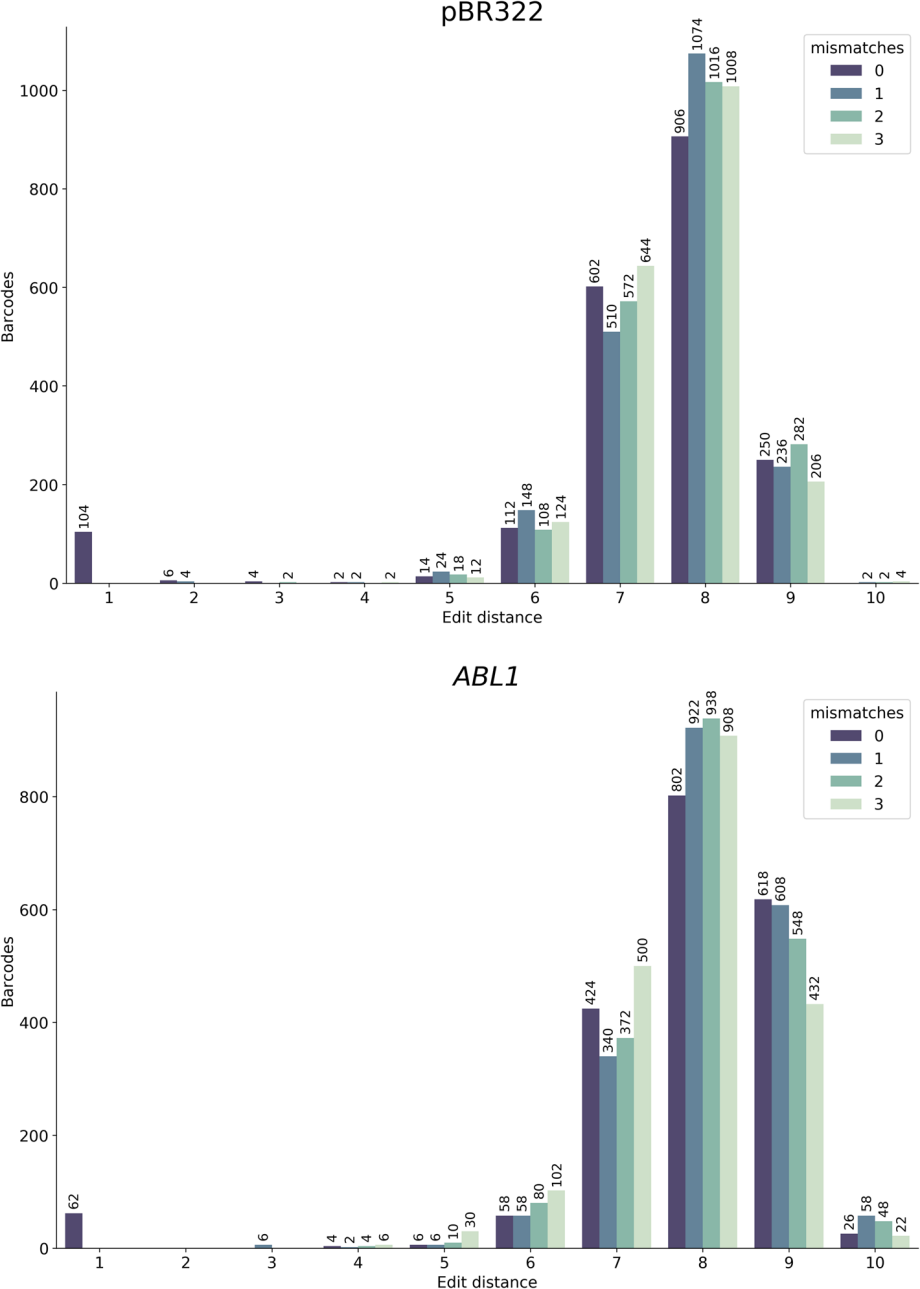
Analysis of inter-barcode edit distances with and without error correction. A randomly selected set of 2000 barcode combinations were compared against all barcodes in *ABL1* and pBR322 datasets before (0 mismatches) and after (1, 2, or 3 mismatches) error correction. The Y-axis is the number of barcodes and the X-axis is the edit distance. For example, without error correction 104 barcodes differ by one nucleotide from barcodes in the entire pBR322 dataset. Error correction completely abolishes barcodes with 1 nucleotide difference

**Fig. 3:**
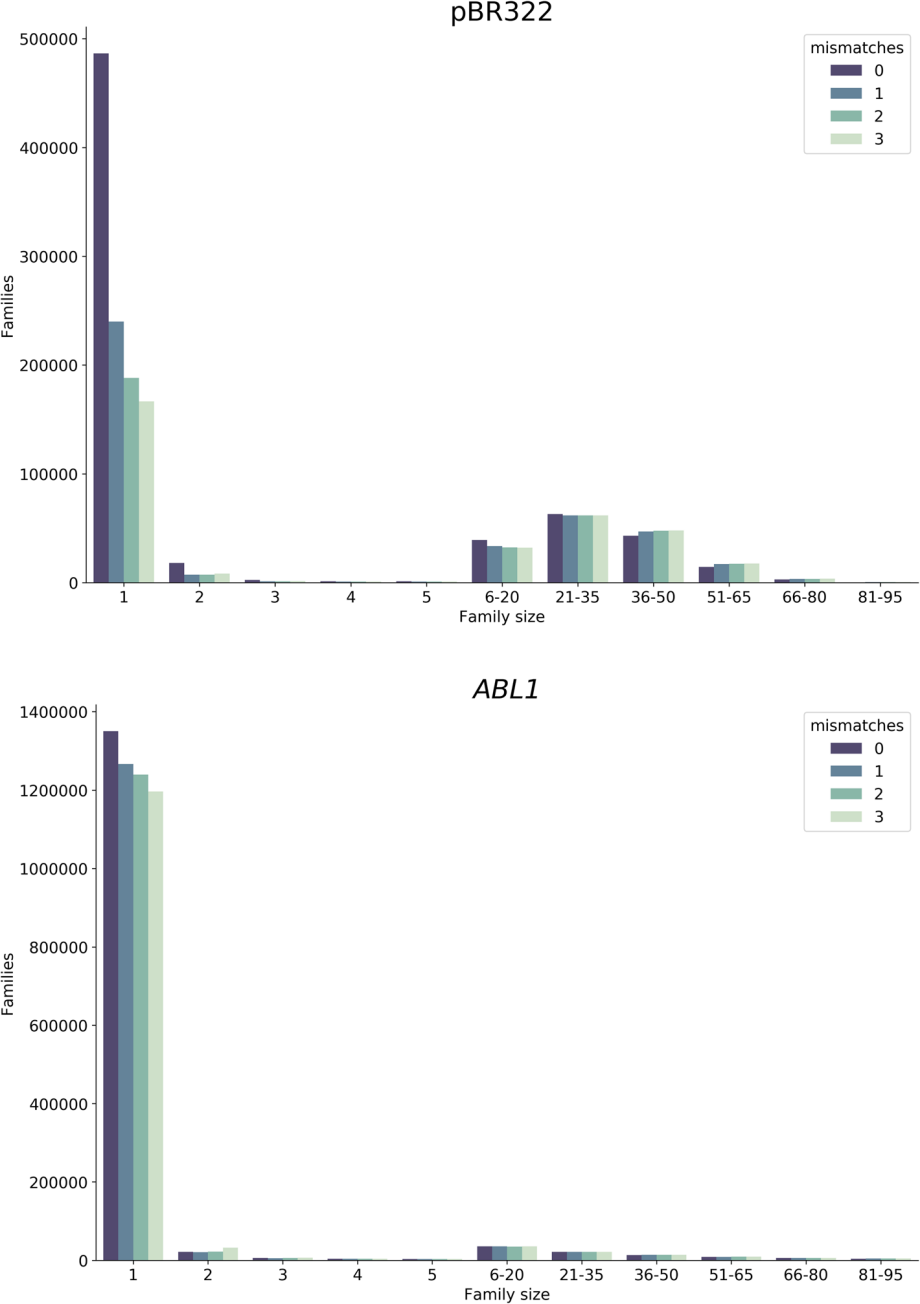
Distribution of SSCS family sizes with and without error correction. Single stranded consensus sequences (SSCS) are created when reads with identical barcodes are bundled together. A common practice requires at least three reads with identical barcodes to form a SSCS. Without error correction (0 mismatches) there is a striking abundance of singletons: single reads with a barcode that is different from all other barcodes in the sample. Applying error correction with progressively higher number of allowed mismatches (from 1 to 3) significantly decreases the number of singletons by re-uniting them with other reads

To check the validity of this reasoning, we analyzed barcodes from the two datasets mentioned above—*ABL1* and pBR322. To do so we trimmed barcodes off of all sequencing reads generating a list of 12 + 12 barcode combinations. We then selected 1000 random combinations from this list to reduce the time required for subsequent computation (out of 1,492,080 and 671,290 barcode combinations for *ABL1* and pBR322 datasets, respectively). Because duplex reads derived from different strands of the same original fragment contain the same two 12 mers but in different order (Fig. 1) there is a total of 2000 tags we chose for this analysis. This is because for each 12 + 12 bp combination *ab* (*a* is the first 12-mer and *b* is the second) we also selected a complementary arrangement *ba.* Next, we compared each of the 2000 tag combinations (concatenated into a single 24 nucleotide fragment) against all other tag combination in the entire dataset. At each comparison we calculated the number of differences (edit distance). Results of this analysis are given in Fig. 2 (dark blue bar “0 mismatches”). One can see that ~ 100 barcode combinations (62 and 104 for *ABL1* and pBR322, respectively) out of 2000 tested have counterparts that differ by a single nucleotide, a difference that is likely introduced by a sequencing and/or PCR error. Because of this difference reads with error-containing tags will not be included into families during the standard duplex data analysis and will be effectively lost. Figure 3 (dark blue bars “0 mismatches”) illustrates this point. Here we sorted all combinations of barcodes in lexicographic order and counted the number of times each combination appears in this list. There is a striking abundance of combinations that appear only once. Such singletons cannot be used in an analysis and are discarded. However, they account for a large fraction of total sequencing output of the two experiments. Our goal was to see if barcode error correction can reduce such waste by recovering reads forming singleton families and returning them into analysis.

### Barcode error correction increases yield

Forming families of reads descended from the same original fragment requires grouping reads by barcode (Figs. 1 and 3). This is straightforward when no sequencing errors are present and can be done by simple lexicographic sorting. Yet as we have shown in the previous section, errors are widespread and this eliminates sorting as a legitimate analysis strategy. An alternative approach will involve performing all-versus-all comparison of all barcodes to identify those that differ by just a few nucleotides and further checking them to see if they are potentially derived from the same DNA fragment with differences being introduced by PCR or sequencing errors. The challenge is that the all-versus-all comparison has *O*(*n*^2^) time complexity and thus is prohibitive as a routine analysis strategy. There are several tools that approach this problem in different ways. The most common strategy is to reduce the search space by first aligning the raw reads to the reference genome. One can then consider barcodes of only those reads that align to one region of the reference. This does not change the time complexity of the search, but reduces the search space from the millions of barcodes in the entire sample to the dozens that may be aligned to a particular genomic location. Several tools are available which use this strategy [1, 12, 15]. However, reference-based approaches are inevitably biased and it was our main impetus to avoid the use of a reference sequence [13]. Alternatively, a strategy implemented in MAGERI, a tool which does not require a reference sequence to form consensus sequences, is able to perform efficient barcode error correction with the use of a custom seed-and-extend alignment algorithm [10, 11]. However, it only forms single-strand consensus sequences, not the duplex consensus sequences required in our analysis.

To overcome these limitations we have adopted Burrows-Wheeler *k*-mer indexing implemented in Bowtie [2] to quickly perform all-versus-all comparison of duplex tags. We are using the original version of Bowtie (not Bowtie2) that was optimized for very short reads. Specifically, we create an FM-index for all barcodes in the sample and then align individual barcodes (as if they were reads) against that index. Results of this alignment are represented as a graph where each vertex corresponds to a barcode. An edge is drawn between two vertices if an alignment exists between two barcodes. An alignment should have a user-defined minimum mapping quality and maximum edit distance, with default values set to 20 and 1, respectively (in the discussion below we vary edit distance values from 1 to 3). The resulting graph contains a large number of disconnected clusters, each of which theoretically represents a single barcode together with all its derivatives created due to PCR and sequencing errors. A correct barcode can therefore be chosen by picking the vertex whose barcode tags the highest number of reads. To assess the effectiveness of this error correction strategy we have developed a tool for producing simulated DS data (see Methods). Using this simulator we produced 400,000 duplex reads and analyzed them using our error correction approach. We then proceeded to calculate how many families (and thus, DCSs) were added to the analysis because of the correction. This increase in yield—the most important consequence of error correction— was substantial. The 400,000 simulated duplex reads produced 43,344 DCSs without correction. Running error correction by setting edit distance to one, two, or three mismatches resulted in 52,896, 53,420, and 53,454 DCSs, respectively. This constituted a 23% increase in yield (at three mismatches) compared the an uncorrected analysis. Effectively the error correction algorithm “shrinks” the pool of singletons (family size, FS, of 1) by reuniting them with families containing correct barcodes, increasing the likelihood that a group of reads surpasses the minimal member number (family size [FS] ≥ 3) for calling a SSCS.

Next, we proceeded to test our approach on real duplex sequencing datasets we used above. We specifically explored if tag error correction improves the number of consensus bases in SSCS and DCS, when allowing for 1, 2, or 3 mismatches in the tags. The results of error correction are summarized in Table 1, Figs. 2 and 3. The error correction decreased the number of singletons (FS ≥ 3) while increasing the numbers of DCSs by re-incorporating singletons into duplex families. This was particularly striking in pBR322 dataset, where the number of DSC increased from 77,164 to 89,513 (Table 1). One can also see that increasing the edit distance during error correction to 2 or 3 did not have such a drastic effect in reducing the number of singletons and increasing the overall SSCS and DCS.

**Table 1:** Effect of error correction on duplex datasets analysis as the number of single strand consensus sequences (SSCS) and duplex consensus sequences (DCS) called after no error correction (0 errors) and error correction with three thresholds of 1, 2, and 3 mismatches allowed

Sample	ABL1				pBR322			
# errors	0	1	2	3	0	1	2	3
SSCS ab	38,493	37,803	37,007	36,280	84,231	81,929	78,481	73,647
SSCS ba	38,202	37,496	36,772	36,080	84,085	81,741	78,234	73,160
DSC	20,745	21,299	22,151	23,180	77,164	80,640	84,359	89,513

### Du novo corrects most barcode errors

With the simulated dataset, we were able to examine the accuracy of barcode correction. Using Du Novo, we corrected the simulated data using three different thresholds for edit distance. Because the dataset was simulated, we were able to compare the corrections made by Du Novo to the ideal set of corrections (Table 2). We can classify these into true and false positives and negatives by considering a correction to be a “positive” and an omitted correction a “negative”. When setting a stringent edit distance threshold of 1, erroneous corrections (false positives) occurred only five times (out of 816,335 corrections). But the tradeoff was that Du Novo was only able to catch and correct 67.28% of barcodes containing errors. Setting a more aggressive edit distance threshold of 3 allowed 77.97% of erroneous barcodes to be corrected, but this caused 2740 barcodes to be wrongly corrected (0.29% of 936,582 corrected). So even with the most conservative threshold, over two thirds of erroneous barcodes are rejoined with their true families, and only 1 in 163,267 of these newly formed families are artifactual.

**Table 2:** Robustness of barcode error correction, measured through simulated data

Edit distance		Positive (%)	Negative (%)
1	True	99.999	67.281
	False	0.001	32.721
2	True	99.983	74.971
	False	0.017	25.033
3	True	99.710.	77.972
	False	0.290.	22.033

The barcodes of 400,000 simulated duplex reads were corrected with Du Novo 2.15 with three different edit distance thresholds: 1, 2, and 3. Corrected and uncorrected barcodes were compared to the original, true barcode sequences. For each corrected barcode, if the correction assigned it to its true family, this was counted as a true positive. Otherwise it was a false positive. Uncorrected barcodes which were not one of the original, true barcodes were counted as false negatives. The rest of the uncorrected barcodes were counted as true negatives. Each family was counted once, rather than each raw read

### New alignment engine improves consensus generation

The first version of Du Novo had a number of limitations resulting in poor performance. It was taking close to 24 h to analyze a single duplex experiment. There were two primary reasons for this: the use of MAFFT aligner and inadequate parallelization strategy for executing multiple consensus generating jobs.

**Fig. 4:**
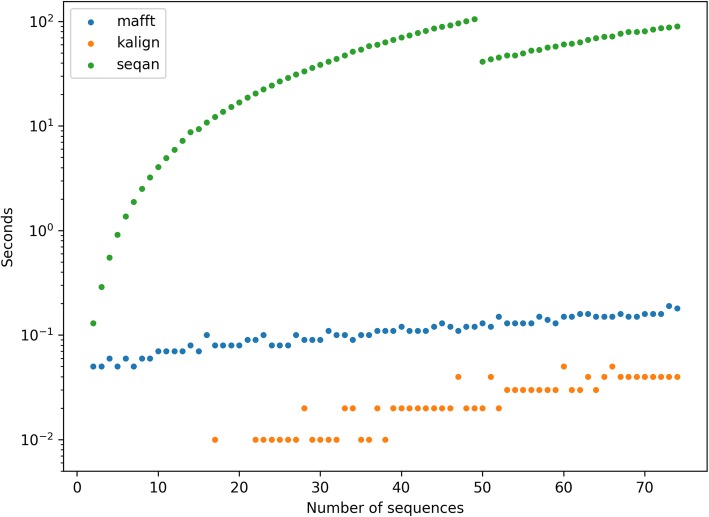
Alignment engine comparison. Comparison of the three aligners tested for use in the Du Novo pipeline

First, we sought to increase the performance of consensus generation step by employing a different multiple alignment tool that can be integrated into Du Novo codebase. We evaluated two candidate tools: SeqAn (https://github.com/seqan/seqan) and Kalign2 [4]. SeqAn is a library of algorithms, including a multiple sequence aligner, specifically written to be incorporated into other genomics tools. Written in C++, it can be compiled and its functions called from Python. Kalign2 is an aligner using the Wu-Manber approximate string-matching algorithm [14] to significantly speed up alignment while maintaining accuracy. Kalign2 is written in C and can also be compiled and called from Python. With some modification, it is possible to communicate with its functions directly from Python, without temporary files. This allows the greatest efficiency and greatest integration into a Python process. SeqAn and Kalign2 were evaluated against MAFFT, the existing algorithm in use by Du Novo. The aligners were tested by performing a multiple sequence alignment on a duplex read family extracted from a duplex experiment sequencing the whole human mitochondrial genome [13]. The family contained 74 reads, 41 single nucleotide substitutions relative to the consensus, and no indels. The number of reads in the alignment was varied from 1 to 74, and the time taken to perform the alignment was measured. Figure 4 shows the results of this experiment. SeqAn was the slowest at all alignment sizes, with the worst performance at handling of large alignments. It took 58× more time than MAFFT at 10 reads, and 427× more at 40 reads. The fastest for all sizes was Kalign2. At 10 reads, it took less than 10 milliseconds. At 30 reads it was 9× faster, but at 60 reads it was only 4× faster than MAFFT. Since the median family size for an ideal duplex experiment is only around a dozen reads, Kalign2’s advantage is significant and we chose it as the default alignment engine for Du Novo.

### Smarter parallelization improves speed

Du Novo uses the multiprocessing Python module for parallel processing. In order to maintain the ordering of the aligned families, the old algorithm would start *N* alignment jobs in parallel, then check each job in order for results. This created a bottleneck at the slowest job: the *N* jobs would take as long as the slowest one. The new algorithm maintains a queue of jobs executing or waiting to be executed. In order to maintain ordering the algorithm keeps an ordered list of submitted jobs. It fills the queue, then begins processing outputs in order of submission. This still requires waiting for all jobs in a batch to finish before continuing, but to reduce the bottleneck, the queue is made larger than the number of available workers. As soon as a job finishes and a worker is freed, it begins work on a new job. This lets new jobs run on CPUs freed by the fastest jobs while the slowest job is still running. If *W* is the number of workers and *M* is a multiplier such that *M* × *W* is the queue size, then a single batch of *M* × *W* jobs will take less time than *M* batches of *W* jobs. Diminishing returns occur as *M* grows, so *M* is set to eight by default. To show the combined effect of the change in alignment and queueing algorithms, Du Novo 2.15, using Kalign2 and the default queue size was compared with Du Novo 0.4, using MAFFT and the old queueing algorithm. Table 3 shows that the combination of the two changes results in an over 9× faster performance at low levels of parallelization. The trend in memory usage is the same as when comparing Kalign2 vs. MAFFT.

**Table 3:** Time and memory usage of different versions of align-families.py, using different multiple sequence alignment algorithms

version	aligner	time/ memory	CPUs					
			**1**	**2**	**4**	**8**	**16**	**32**
0.4	MAFFT	time (seconds)	28,638	15,769	8912	5173	3038	1747
2.15	MAFFT		28,754	14,282	7079	3463	1686	854
	Kalign2		4731	1777	945	600	381	246
0.4	MAFFT	memory (MB)	23,704	12,299	6622	3755	2284	1602
2.15	MAFFT		23,927	12,599	6850	3985	2541	1810
	Kalign2		24,648	23,220	12,408	6668	3781	2327

At low levels of parallelization, Kalign2 made the process over 8 times faster, with a memory usage less than twice as much as MAFFT. The new algorithm sped up the tool between 1 and 2.05x. Naturally, at higher levels of parallelization, the reduction of the job queue bottleneck made more of a difference. Memory usage appeared to not be affected, which is expected due to the small size of the job queue compared with the rest of memory usage. To attempt to disentangle the effects of the job queueing algorithm from all the other changes between 0.4 and 2.15, the two versions were compared with all parameters set as similarly as possible. In both cases, the number of **--processes** was set to 32 and MAFFT was used as the aligner. Crucially, the **--queue-size** for the 2.15 version was set to be 32, the same as the number of **--processes**. This approximates the bottleneck in the pre-2.0 version of Du Novo’s job queueing algorithm. Comparing the median of 3 trials of each, the wallclock time of 2.15 was 27% higher than that of 0.4. This could be because of the higher overhead in the more complicated parallelization algorithm, or other changes between 0.4 and 2.15

Next we used the simulated dataset to test whether the change in alignment algorithm affects the accuracy of the pipeline. The simulated experiment was the same, but with 40,000 fragments generated instead of 400,000. Because the input was one homogenous sequence with no minor variants, any differences from the input must be due to incorrect consensus base calls. Using the previous multiple sequence aligner, MAFFT, resulted in an error rate of 0.00563 differences per output base (Table 4). Using Kalign2 instead resulted in 0.00561 differences. Adding barcode error correction improved this figure slightly to 0.00525 while also increasing the yield. The standard pipeline published by Loeb et al. [8] was also compared, resulting in 0.0114 differences per output base.

**Table 4:** Effect of aligner on “correctness”

Method	Aligner	Barcode error correction	Errors per base
Du Novo	MAFFT	Uncorrected	0.563%
Du Novo	Kalign2	Uncorrected	0.561%
Du Novo	Kalign2	Corrected	0.525%
Loeb	N/A	Uncorrected	1.140%

## Conclusions

In this manuscript we have introduced an error correction approach to the analysis of duplex sequencing data. This allows correcting errors in barcodes thereby reducing data loss and increasing the yield of duplex sequencing experiments. We made a number of other improvements including a new alignment engine and advanced parallelization. Finally, we made the new software readily available to a wide audience of users. To achieve this goal we are distributing Du Novo in three complementary ways:

- Interactive pipeline at http://usegalaxy.org. Here users can upload datasets of any size and process using the complete Du Novo pipeline to produce SSCS and DCS sequences. The Galaxy system contains all tools for downstream processing including mapping and variant calling. To help users effectively use our system we have developed a detailed step-by-step tutorial that can be found here: http://bit.ly/dunovo-tutorial.
- Bioconda package. Du Novo code relies on a number of software components that need to be installed before the tool can be used. Conda package eliminated the need to install these dependencies by automatically installing all components using **conda install dunovo** command (see http://bit.ly/dunovo-bioconda).
- Source code for the package can be found in GitHub at https://github.com/galaxyproject/dunovo. It is distributed under Academic Free License.

## Methods

### Barcode error analysis

The edit distance quantifies similarity or dissimilarity between two DNA sequences of equal length by calculating the number of differences between them:

$$D_{i,j}=\sum_{k=1}^{n}{[X_{ik} \neq X_{jk}]}$$

D_*i,j*_ is the number of sites where X_*i*_ and X_*j*_ do not match, *k* is the index of the respective site out of a total number of sites *n*. The input data was in tabular format organized into family size, the sequence of the tag, and the direction of the tag in the SSCS (*ab* = forward or *ba* = reverse). Each tag represents a family of paired-end sequences forming SSCS. Since the whole dataset contained more than one million tags, the comparison of all tags was computationally too demanding. Thus, we parallelized the algorithm and only selected 1000 random tags from the data set and compared them to the whole dataset to estimate the minimum edit distance between tags. A sample of 1000 tags gives a very similar estimate to the edit distance estimated for a sample of 10,000 and ~ 130,000 tags.

### Error correction

The script **baralign.sh** performs an alignment of all barcodes against themselves (all scripts mentioned in this section can be found at https://github.com/galaxyproject/dunovo). First, it extracts all unique barcode sequences (concatenations of *a* + *b* tags) as FASTA sequences. Then it indexes them, along with their reversed (*b* + *a*) versions, with bowtie-build and aligns them to the index with **bowtie -v 3 --best -a**. This alignment is then read by **correct.py**, which uses the networkx module to construct graphs where each vertex is a barcode and each edge is a high-quality alignment between two barcodes. The definition of a high-quality alignment is configurable and based on the MAPQ mapping quality, the edit distance given by the NM tag, and the distance between the aligned starting positions of the two barcodes. The default values for these filters is 20, 1, and 2, respectively. Then, for each graph, a “correct” barcode is chosen by one of two methods. The default method is to choose the barcode which tags the largest number of reads. An alternative is to choose the barcode with the most edges to other barcodes.

### Generating simulated duplex data

To validate the effectiveness of our approach we have first applied it to a simulated duplex sequencing dataset generated with a duplex sequencing simulator developed to test the correctness of the Du Novo algorithms against known duplex sequencing behavior and sources of errors. It simulates an entire duplex sequencing experiment but taking a reference genome sequence as input, randomly fragmenting it, adding random barcodes to the ends of these fragments, simulating PCR and sequencing errors to produce a set of simulated duplex reads. To randomly fragment the reference sequence, it uses **wgsim** (https://github.com/lh3/wgsim) in error-free mode (options **-e 0 -r 0 -d 0**), using the **− 1** option to set the length of the fragments. Then it simulates random oligomer synthesis to produce duplex barcodes using a uniform 25% probability for each base. It concatenates these oligomers, along with a constant linker sequence, with the fragment sequence to produce starting fragments. These simulated tagged fragments then undergo in silico PCR in order to introduce amplification errors. First, a family size is chosen from an empirical distribution observed in a previous duplex experiment. Then, the phylogenetic tree relating these reads is generated. For a family size of *n*, the process starts with *n* reads at the root node representing the original fragment molecule. Each read is randomly assigned to a daughter molecule with 50% probability. Then the process repeats with each daughter, using the number of reads assigned to the daughter instead of *n*. Because amplification efficiencies decline as PCR cycles continue, the probability of replication starts at 1 and is divided by 1.05 each cycle, a realistic value compared with observed reactions [3]. Once a tree is generated, errors are simulated at each node and propagated to their descendents. Then, sequencing is simulated, also with errors, and reads are output. A log of the errors is also saved, in order to allow checking results against the “truth”. Unless noted otherwise, simulated data presented here were generated with a sequencing and PCR polymerase error rate of 0.001 errors per base. Twenty five cycles of PCR were simulated, the fragment lengths were set to 400 bp, and the read lengths to 100 bp. Using this approach we have generated a dataset containing 400,000 simulated duplex reads and applied our error correction strategy.

### Du novo 2.0

The basic algorithms in Du Novo 2.15 remain as described in [13], except the addition of barcode error correction, the Kalign2 multiple sequence aligner, and the replacement of the parallel job queueing algorithm. In all experiments described here, the threshold required to form a consensus base (**make-consensi.py**’s **--cons-thres**) was 0.7, 3 reads were required to create a consensus sequence (**−-min-reads**), and a PHRED score of at least 25 was required to count a base toward the consensus (**−-qual**).

When consensus reads were filtered, the script **trimmer.py** was used from the **bfx** directory of Du Novo’s distribution. Unless noted otherwise, the script was set to remove the 5′ end of reads when the proportion of N’s in a 10 base window exceeded 0.3 (**−-filt-base N --window 10 --thres 0.3**). If either of the reads in a pair was trimmed to less than 75 bases, both were removed (**−-min-length 75**).

## Data Availability

All software is freely available under a non-restrictive AFL 2.0 license. It can be accessed via Galaxy system at https://usegalaxy.org or downloaded from GitHub at https://github.com/galaxyproject/dunovo

## Abbreviations

DCS: Duplex Consensus Sequencing
DS: Duplex Sequencing
PCR: Polymerase Chain Reaction
SSCS: Single Stranded Consensus Sequence
